# Recycling Waste Fiberglass by Powder Grinding and Direct Molding of Powders

**DOI:** 10.3390/polym17070987

**Published:** 2025-04-05

**Authors:** Fabrizio Quadrini, Leandro Iorio, Giorgio Patrizii, Denise Bellisario, Loredana Santo

**Affiliations:** 1 Department of Industrial Engineering, University of Rome «Tor Vergata», Via del Politecnico 1, 00133 Rome, Italy; leandro.iorio@uniroma2.it (L.I.); giorgio.patrizii@uniroma2.it (G.P.); denise.bellisario@uniroma2.it (D.B.); loredana.santo@uniroma2.it (L.S.); 2 Department of Human and Social Sciences, University of Mercatorum, Piazza Mattei, 10, 00186 Rome, Italy

**Keywords:** fiberglass, recycling, direct molding, compression molding, thermosets

## Abstract

Direct molding is a compression molding process of thermoset particles without the addition of any linking agent or binder. It is suitable for recycling end-of-life fiberglass or other waste from the manufacturing of fiberglass products. In this study, for the first time, the feasibility of recycling waste fiberglass powder, collected from an industry, is shown in the case of a vinyl ester matrix. Powders have been directly molded, without any pre-treatment such as sieving, to manufacture small samples for four-point bending tests. Supplied powders have been characterized by microscopy and thermal analysis. Its size distribution has been evaluated by sieving, and the amount of resin by burning test. Samples have been compression molded in an eight-cavity mold and have shown good homogeneity and surface aspect. The average density of the recycled fiberglass is 1.23 g/cm^3^, the bending strength 28 MPa, the elongation at break 1.6%, and the elastic modulus 1.9 GPa, with low dispersion (7% at maximum). Surface analysis has shown a rough surface and the presence of embedded glass fibers into the agglomerated fiberglass. Results show that waste powders from secondary processes of fiberglass manufacturers, such as surface grinding, may provide secondary raw materials for the production of molded parts without mixing with virgin substances.

## 1. Introduction

The production of fiberglass in Western Europe has reached the impressive amount of 1 million tons per year. According to the European Commission, about 25% of waste fiberglass ends up in landfills, with significant environmental concern posed by this type of material. Nowadays, thermoset fiberglass is a common material which is widely used in many applications, from boats to electrical appliances, sport devices, and construction. It comes from the combination of a polymeric thermosetting matrix, mainly polyester, with glass fibers in different configurations (fabrics, unidirectional tapes, mats). Fiberglass manufacturing is very easy and cheap, particularly for large products, but its end-of-life is still problematic because of the difficulty of separating the matrix from the fibers, and the poor value of the resulting secondary materials.

The main approach in fiberglass recycling is still shredding, as fiberglass products are very often large, and possibly powder grinding, for further reprocessing or energy recovery. In 2023, Lekshmi et al. evaluated the impact of boats made of fiberglass along the coast of Kerala, India, because of their abandonment and burning [1]. They discussed about the use of pulverization to reduce volumes and therefore costs for transportation. In the same year, Rizzello et al. [2] pointed out that fiberglass components have mechanical, electrical and fire resistance characteristics that make them well-suited for application in power distribution grids, but the thermoset polymers that constitute them are not recyclable, significantly impacting their end-of-life management. A possible solution involves choosing recycling techniques that increase the amount of recycled material in the new compound while reducing the energy impact from processing the material.

**Figure 1:**
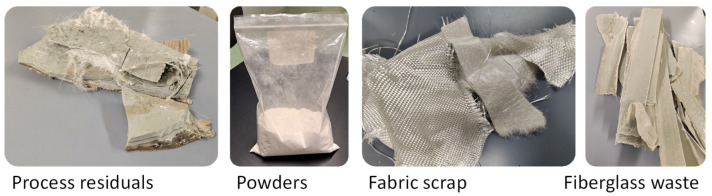
Typical industrial waste from the manufacturing of fiberglass, including powders.

In the past, many different technologies have been studied for recycling end-of-life products or industrial waste of manufacturers (Figure 1), but most of them have been abandoned. In 1997, Yoon et al. showed a chemical recycling technology for fiberglass, by using propylene glycol for extracting reactants which can be cured with the addition of maleic anhydride [3]. In 1998, Kennerley et al. discussed fiber recovery from thermoset composite waste with a fluidized bed process [4]. The inorganic solids, like metal inserts, sink in the fluidized bed and can be removed, while organic contaminants, like mineral oils, are volatilized with the polymer matrix. The same authors also improved this process [5], but this application has been poorly developed. In fact, for this aim, pyrolysis seems to be more effective. In 2003, Cunliffe et al. [6] proposed the use of a static-bed reactor with a range of temperatures from 350 to 800 °C to pyrolyze different composites, including fiberglass. In 2004, Kaminski et al. evaluated the use of a fluidized bed instead of a static-bed reactor for pyrolysis [7]. Apart from the adopted technology, pyrolysis showed some issues related to the cost of the equipment, and the energy loss during the recycling process. Furthermore, pyrolysis needs secondary processes, like the cleaning of glass fibers or burning of pyrolytic oil, to take advantage of the recycling process.

There are several review studies that show the important role of mechanical processes in fiberglass recycling and the potential impact of technologies such as pyrolysis. Today, the main recycling strategy for fiberglass recycling remains the mechanical process (pulverization) whereas thermal (combustion, pyrolysis, fluidized bed) or chemical (solvolysis) processes may be considered as secondary alternatives [8]. Mechanical recycling starts with a size reduction into smaller pieces, making it easier to remove metal inserts while making transportation simpler. The material is then slowly crushed in a milling or grinding machine into smaller pieces. The result of mechanical recycling is a mixture of resin, filler (if present), and fibers with different aspect ratios that may have to be further sieved. The main advantages of mechanical recycling are the industrial scalability and the low environmental impact. The challenge is evaluating how these secondary materials can be efficiently reused to manufacture new products. In fact, all these recycling technologies at the state-of-the-art, from grinding to pyrolysis, do not produce new parts but inert fillers for following processes. Generally, an organic matrix is added to make new parts, such as in the extrusion or injection molding of thermoplastics.

In 2010, Guo et al. showed that fiberglass ground powders, a mixture of resin powder and glass fibers reclaimed from pulverized waste printed circuit boards (PCBs), can be used as a partial substitute for wood flour in the production of a modified phenolic molding compound [9]. The results show that the incorporation of fiberglass powder as a filler into the molding compound enhances its thermal stability. In 2015, Sun et al. proposed to reuse the fiberglass recovered from waste PCBs for a noise reduction application [10]. They found that the great porosity of the ground fiberglass resulted in excellent sound absorption across a wide range of frequencies.

Another interesting example was discussed by Cozzarini et al. in 2023 [11]. This research examined a lightweight thermal and acoustic insulation material, produced starting from a hydrogel-based mixture composed of renewable biopolymer and fiberglass waste powders.

Rahimizadeh et al. in 2019 evaluated different strategies for recycling and reusing the fiberglass rotor blades of wind turbines [12]. The authors showed that chemical and pyrolysis processes to recover glass fibers from end-of-life turbine blades are inefficient in terms of commercialization due to the use of hazardous chemicals and/or excessive costs. In their opinion, the only recycling process which has industrial applications is mechanical grinding. They proposed a method to combine mechanical recycling and 3D printing by the Fused Filament Fabrication process, with the aim of improving the mechanical performance of 3D printed components. Results showed that the mechanical properties of the recycled composite parts with shredded particles were comparable to those observed for parts made of pyrolyzed fibers, without the issues introduced by the pyrolysis process. Dealing with end-of-life turbine blades, Revilla-Cuesta et al. proposed a novel material, called Raw-Crushed Wind-Turbine Blade, obtained from the recycling and non-selective crushing of wind-turbine blades composed of fiberglass composite fibers, polyurethane and balsa-wood particles [13]. It serves as concrete fibers and aggregates, according to its physical and microscopic characterization. In a subsequent study [14], the same authors evaluated the water-transport and porosity behaviors of these concretes through different tests. As a general consideration, mechanical recycling is easy but the properties of the final recycled parts, as well as their manufacturing technologies, depend on the adopted new matrix, which is generally a virgin material.

A recent innovation aims to manufacture recycled products by using 100% of fiberglass waste powders [15]. This technology, namely “direct molding”, consists of the compression molding of fiberglass powders without the addition of a linking agent or binder [16]. In fact, pulverizing through mechanical grinding or machining is an effective way to give new reactivity to waste recycled powder. Broken bonds on particle surfaces can act as polymerizing sites, which are combined with intrinsic residual reactivity of waste fiberglass. Therefore, end-of-life products can be pulverized and “directly molded” to make new products. As an alternative, waste powders can be recovered from fiberglass manufacturers (Figure 1), being a typical residual product from such finishing or cutting operations [17]. In this study, industrial powders were collected from industry and used to show the feasibility of this new recycling strategy, without any additional powder treatment or virgin material. For the first time, powders from the finishing of large parts for marine application were compression molded as collected. Another novelty is that vinyl ester matrix particles were reprocessed whereas previous studies were carried out on polyester [15,16,17]. These powders are never considered for recycling because of their contamination and low residual value. Direct molding can be a way to manufacture new parts on site for internal uses, such as inserts for new molds.

**Figure 2:**
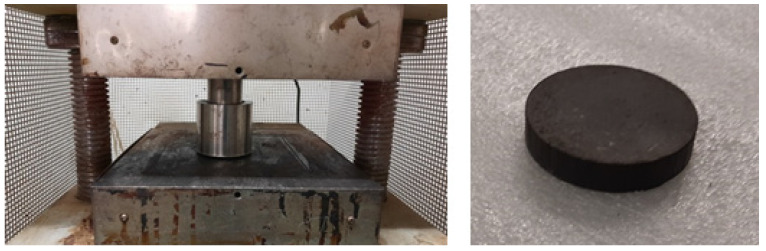
Direct molding of waste powders from the machining of carbon fiber reinforced composites.

Thermoset particles can be aggregated by using “direct molding” and this study is another confirmation of this technological principle. However, in scientific literature, there are still few contributions on this topic, above all in the case of rigid thermoset particles [15,16,17]. In the current experimentation, for the first time, a vinyl ester resin has been used and successfully recycled, in spite of the high content of inert fibers (about 40 wt%). The concept of direct molding can be applied to many other waste powders. For example, in Figure 2, the case of direct molding of powders from machining of carbon fiber reinforced epoxies is shown. In this case, a disk has been molded with similar process parameters of the current study.

In terms of industrial use, the appearance of the recycled sample is an important aspect, because it allows the use of the material without a protective coating, and it increases the technological perception of the recycled material. Direct molding may provide smooth and functional surfaces, depending on the organic content of the waste powder. It is not possible that all waste powders or products can be used to make new recycled parts.

## 2. Materials and Methods

### 2.1. Powder Supply and Characterization

In this study, fiberglass powder was recovered from the waste of an industrial partner (Aviorec srl, Anagni FR, Italy), which specializes in the production of composite parts for the aeronautic, automotive and defense industries. The powder derives from finishing operations on large panels by grinding. The original fiberglass had a vinyl ester resin matrix, cured at room temperature. To characterize the supplied powder, a stereo (Leica S9i, by Leica Mycrosystems, Wetzlar, Germany) and an optical (Leica DMIRM) microscope were used. Moreover, the powder size distribution was evaluated by sieving using four sieves with mesh sizes of 50, 100, 170, and 270. Subsequently, the organic content was estimated using a burning test in an oven (Nabertherm B170, by Nabertherm Gmbh, Lilienthal, Germany) for 2 h at a temperature of 600 °C. Differential scanning calorimetry (by Perkin Elmer DSC6 (Perkin Elmer, Waltham, MA, USA)) was also carried out to evaluate the glass transition temperature of the matrix and possible additional transitions. A double scan from room temperature to 150 °C, at the cooling rate of 10 °C/min rate, was also performed for this aim.

### 2.2. Direct Molding of Recycled Fiberglass

**Figure 3:**
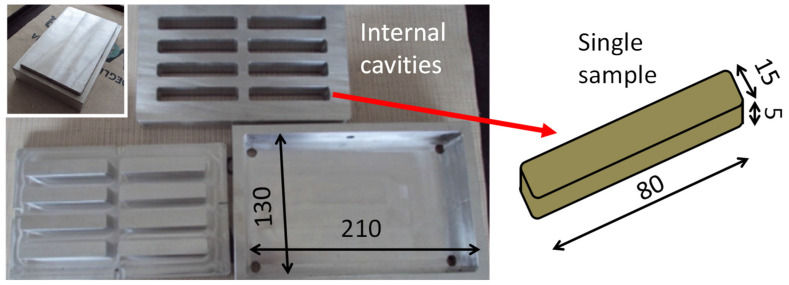
Mold for recycled sample fabrication and nominal sample size.

Samples of recycled composites were manufactured by compression molding of the waste powders (namely “direct molding”) without any powder pre-treatment or additional virgin material. Rectangular samples were molded using an aluminum alloy mold which consisted of three elements: a shaped upper punch, a bottom plate, and an intermediate plate with eight cavities (30 mm in depth), matching the shaped punch (Figure 3). This configuration was chosen to simultaneously extract eight samples of 80 × 15 × 5 mm^3^ after molding. A release film was inserted between the bottom and the intermediate plate to prevent sticking.

Compression molding was carried out in a hot parallel plate press by applying three-bar pressure (0.3 MPa) at a temperature of 250 °C for 60 min. The long molding time was due to the fact that the mold was initially at room temperature because of the laboratory procedure. After molding, the mold was removed from the press and left to cool to room temperature before sample extraction.

**Figure 4:**
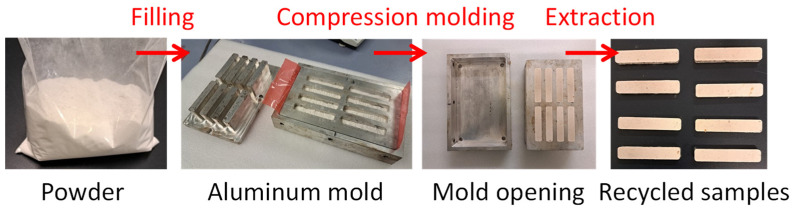
The direct molding process to recycle fiberglass waste powders.

High temperatures are necessary to increase resin mobility and, therefore, to enhance particle agglomeration. The temperature is close to the resin degradation temperature, at which the possibility of polymer chain breakage is high but also the possibility of creating new links increases [17]. The pressure, instead, is important to have contact between particles during the powder re-agglomeration process. The molding process lay-out is shown in Figure 4.

The density of the recycled samples was calculated after weight and size measurements. The average thickness of the 8 molded samples was 4.9 ± 0.2 mm, with a very low dispersion (about 4% of the mean value).

### 2.3. Sample Testing

**Figure 5:**
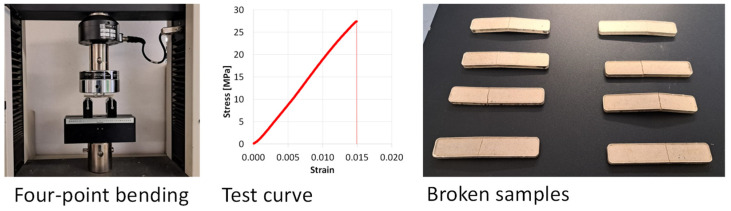
Mechanical testing of recycled samples by four-point bending.

Mechanical testing was carried out by four-point bending in a universal material testing machine (MTS Insight 5 (MTS Systems Corporation, Eden Prairie, MN, USA)) [18]. The distance between the loading pins was 20 mm, while the distance between the supporting pins was 60 mm. This configuration was chosen because of the brittle nature of the recycled samples, which behave like agglomerated materials. The set-up configuration of the bending test is shown in Figure 5, together with a typical stress–strain curve and the picture of all the broken samples. Despite their brittle nature, after breaking, the samples did not separate into two halves, because of the presence of small glass fibers across the failure surfaces. At the end of the test, the bending strength, the elongation at break and the bending modulus were extracted from each stress–strain curve. Finally, the surface aspect of the molded samples was investigated using a digital microscope (Hirox HRX-01, Hirox Europe, Limonest, France).

The topography of the molded surfaces was analyzed as well as the fracture surfaces of the broken samples. Roughness parameters were evaluated by contact profilometry (Talysurf CLI 2 by Taylor-Hobson, Leicester, UK).

Roughness parameters were extracted from five different samples: 16 profiles were acquired from each sample on an area of 6 × 3 mm^2^, with a resolution of 1 µm along the x-axis, 200 µm along the y-axis and 10 nm along the z-axis.

## 3. Results and Discussion

**Figure 6:**
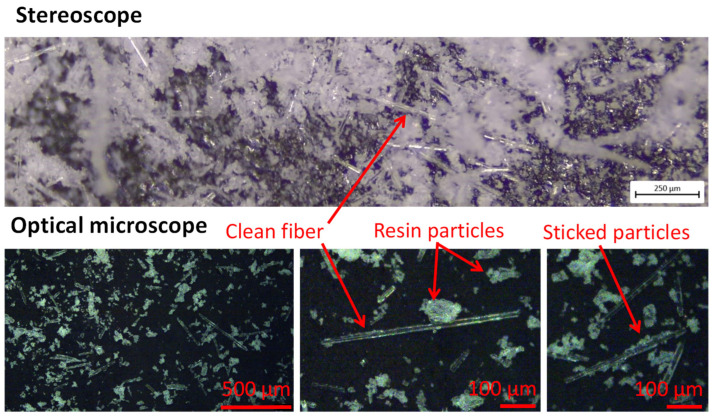
Powder characterization by stereoscope and optical microscope.

**Figure 7:**
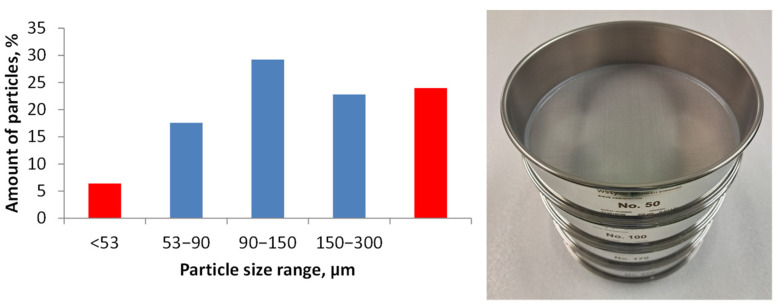
Size distribution of the ground powder by sieving.

In Figure 6, some pictures of the supplied powder from a stereoscope and an optical microscope are shown. The presence of a mixture of free particles and short glass fibers is evident. Free fibers generally have a length in the range of 100–300 µm, but single fibers may reach 500 µm. Moreover, they may be fully clean or have residual resin particles on the surface. The particle size distribution was measured by sieving and is shown in Figure 7; 70% of the particles ranged between 50 and 300 µm, with an average of 120 µm. The size distribution of Figure 7 is reported with size ranges as it depended on the mesh of the adopted sieves.

The average amount of resin was extracted from the burning test and was found to be about 40.6 wt%. As glass fibers are inert, powder re-agglomeration depends only on the waste matrix. Deeper compositional analyses and powder characterization were not carried out because of the intrinsic non-homogeneity and contamination of the collected powder, coming from a finishing operation of a large marine part.

High fiber contents are not recommended for this recycling technology, whereas small amounts of fibers help in increasing the toughness of the recycled samples.

**Figure 8:**
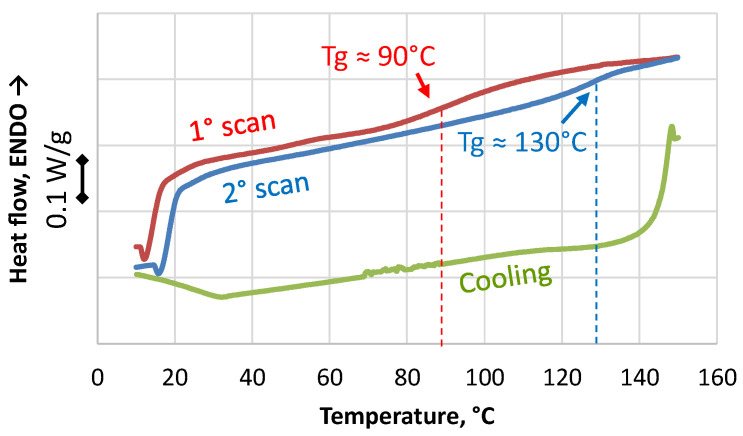
DSC scans of the supplied waste powder: first scans.

The residual reactivity of the powder was analyzed by DSC (Figure 8). In the first scan, the powder softened, and its polymerization could be re-activated at a high temperature. The extent of the residual reactivity depends on the initial cure level of the matrix, which depends, in turn, on the manufacturing process of the virgin composite. In the presence of room temperature curing, the residual reactivity can be significant, but it is difficult to identify a cure peak in the DSC thermogram. Nevertheless, it is possible to infer the extent of the residual reactivity by the shift of the glass transition temperature of the waste matrix between the first and the second scan. In this case, as shown in Figure 8, it shifted from 90 °C in the first scan to 130 °C in the second. Because of the absence of a reaction peak, the extent of the residual reactivity cannot be measured as well as its possible impact on powder re-agglomeration. The cooling stage, between the first and the second scan, showed a glass transition comparable to the second scan, therefore it is reasonable to assume that all the residual reactivity had been spent in the first sample heating.

**Figure 9:**
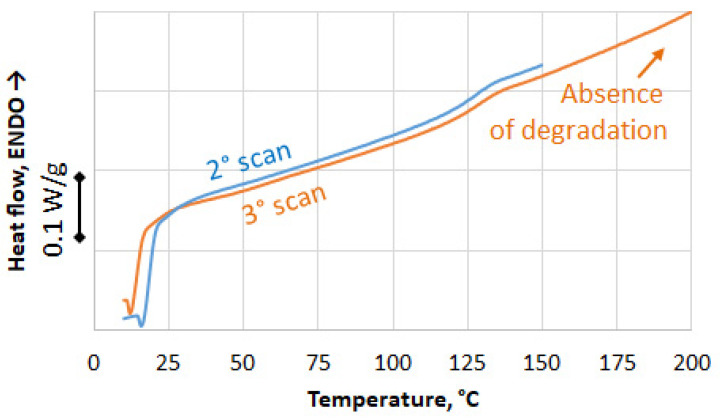
DSC scans of the supplied waste powder: comparison between second and third scan.

To take advantage of the residual reactivity, a molding temperature of 130 °C could be sufficient but this mechanism is not the only one in “direct molding” and a temperature of incipient degradation must be approached. In Figure 9, the second and third scan of the powder are compared. They are superimposed well; therefore, all the residual reactivity was spent in the first scan, and degradation did not occur during the second scan. The third scan was extended up to 200 °C and degradation was still not visible. For this reason, the temperature of the parallel plates of the molding press was set to 250° C, to provide possible incipient degradation and to compensate for the initial cold state of the mold, which was at room temperature at the end of filling.

This strategy seems to be successful, as an acceptable level of agglomeration was reached. Thanks to the small size distribution, the surface appearance of the molded samples was good (Figure 5).

After the molding process there was no release of particles from the surface, and it appeared shiny, intact and consistent. As shown in Table 1, the average density of the samples was 1.23 ± 0.06 g/cm^3^, lower than common fiberglass, which is about 1.5 g/cm^3^. However, the collected powders were produced during the finishing of fiberglass parts when only the external skin of the laminate was involved, therefore a precise estimation of initial density and the final porosity was not possible.

Generally, the filler content is lower in proximity with the laminate surface where a gel coat can be also applied. It is reasonable to assume that the samples are characterized by high porosity because of the agglomeration mechanism of the direct molding process too, where only the particle surfaces are involved. This porosity can affect also the mechanical properties.

The results of the four-point bending test are reported in Table 1. The average bending strength was 28 MPa, but a maximum of over 30 MPa was measured on one sample.

The average elongation at break was 1.6%, with a maximum of 1.7%, measured on the same sample with the highest strength. The bending modulus was 1.9 GPa. Dispersion was generally low, being 7% for strength, 6% for the elongation at break, and 5% for the modulus. Therefore, the molding conditions were uniform enough along the eight samples, which reached a comparable level of agglomeration and similar mechanical properties. The direct molding of elastomers is simplified by the deformability of the particles under consolidation pressure, and the final porosity may be reduced. In the case of rigid particles, their deformability is low, and the contact between particles during molding is poor. Higher porosity and lower performances are expected because of this limitation in comparison with elastomers (such as rubber from tires) [19]. Particles soften at the molding temperature, and this mechanism improves agglomeration, but it is not optimal. It is fundamental to have a good match between the poured powder, at the end of filling, and the punch surface, otherwise only these zones in the recycled part will be well agglomerated (if not over-pressed), whereas other zones will be poorly compacted and weak. In this context, choosing a mold with eight cavities is a challenge because of the difficulty of putting all the powder surfaces in each cavity at the same level. In the proposed study, this goal has been successfully reached, and all the samples of the same molding operation show similar properties.

Mechanical properties are lower than typical fiberglass, because stiffness and strength of the recycled samples depend mainly on the matrix and not on glass fibers. A comparison with virgin fiberglass was carried out in previous studies where a reduction from 70% to 95% of the bending strength was observed after recycling, depending on the fiber content (up to 40 wt%), [16]. This big difference is related to the different nature of the two materials. Virgin fiberglass is a continuous-fiber reinforced laminate whereas recycled fiberglass is an agglomerated bulk material. Moreover, particle joining in the recycled fiberglass is partial and does not involve the entire particle surface, with consequent high porosity. Nevertheless, the final properties could be sufficient for such uses, being comparable with other agglomerated materials such as plasterboard.

The fracture behavior of the recycled sample is brittle, as shown in the stress-strain curve of Figure 5 but the samples do not split into 2 parts after breaking because of the presence of the fibers. Agglomerated materials from powders are brittle because their strength depends on the particle surfaces and not on their intrinsic properties. A failure always starts and propagates through the particle interfaces.

**Figure 10:**
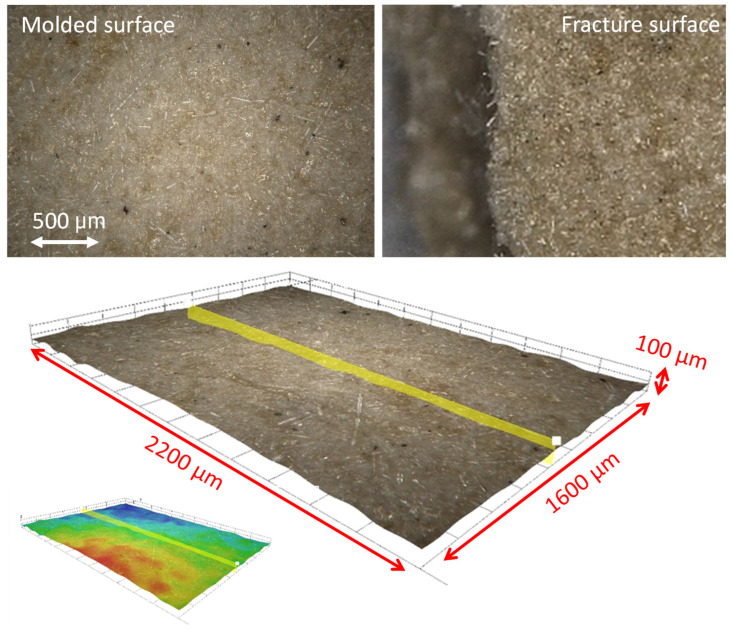
Surface analysis of molded samples.

**Figure 11:**
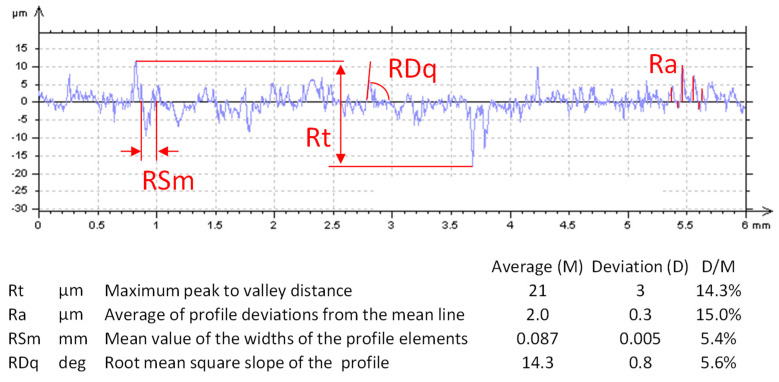
Extraction of roughness parameters from the surface analysis.

Fibers act as bridges between the crack surfaces, adding a minimal toughness even if they do not participate in the material agglomeration, being inert. The presence of small fibers is visible in the surface analysis of the molded samples (Figure 10). They are aligned with the molded surface and emerge from the fracture surfaces. The morphology shows many undulations and irregularities, because of the high surface porosity (estimated to be about 15–20 vol% from density values) and the presence of the embedded fibers. The measurement of the roughness parameters, shown in Figure 11, confirms the irregular aspect of the molded surface. Dispersion on the 5 samples is large but not excessive, by considering the agglomerated nature of the surface, as it is always lower than 15%. The average roughness Ra reaches a value of 2.0 µm on average, therefore the molded surface is relatively smooth. In fact, in the machining of metals, Ra values range from 0.1 µm to 6.3 µm (smooth to rough). This datum means that the part surface is partially flattened by the interaction with the mold surface, which was machined. However, surface open porosity plays its role as Rt, the maximum peak to valley distance, is one order of magnitude higher than Ra. Moreover, the powder size distribution influences the value of RSm, which is close to the maximum of the size distribution curve (Figure 7). Particles are rigid at room temperature, but they are partially shaped during molding, for this reason the value of RDq is smaller than expected. By considering the brittle behavior of the recycled fiberglass, it is expected that sample roughness strongly influences the material strength. Current data are not able to enter into this correlation, and the bending strength is discussed to give evidence of the material agglomeration. In future, surface coating and powder refining will be investigated to increase this value.

## 4. Conclusions

In order to mechanically characterize the recycled composites, samples for a four-point bending test were molded. Making small samples is an advantage for this technology, as the single part receives more heat from the mold walls. By increasing the surface/volume ratio, the agglomeration improves. The obtained properties for re-agglomerated fiberglass (up to 30 MPa of strength and 2 GPa of modulus) seem to be sufficient for such applications, but not substituting the same virgin fiberglass. In comparison with previous studies, when a similar bending modulus was observed, the attained strength more than doubled [16]. The idea is that these materials are recycled by the same factory which produces waste for secondary uses, such as packaging elements, sandwich cores or part protection. For this reason, the collected powder was processed as is, without any treatment. The use of industrial powders has a strong advantage in comparison with powders from grinding end-of-life products, in terms of the homogeneity of the powder characteristics. In fact, factories do not change virgin materials and processes continuously. The collected power may show very similar bulk properties, resin contents and size distributions for long periods, with a positive effect on the reliability of the recycling process.

The main outcome of the current study is that internal recycling of fiberglass waste is possible for the case of a vinyl ester matrix, cured at room temperature. Powders were molded as collected, with all the drawbacks of their contamination and non-homogeneity. For this reason, it was not possible to deepen important scientific aspects such as pressure distribution, powder compaction efficiency, and mold surface effects.

The use of this technology is intended to complement the state-of-the-art technical solutions, comprising burning or pyrolysis, that are preferred in those cases when the powder re-agglomeration is poor. The best recycling scenario remains the on-site production of small parts, such as inserts, to be used by the same fiberglass manufacturer. In this case, direct molding can be competitive with the filling of thermosetting resins, because of the absence of virgin raw materials.

## Figures and Tables

**Table 1:** Properties of the recycled fiberglass.

Density	1.23	±	0.06	g/cm^3^
Bending strength	28	±	2	MPa
Elongation at break	1.6	±	0.1	%
Bending modulus	1.9	±	0.1	GPa

## Data Availability

All experimental data are reported in the manuscript.

## References

[1] Lekshmi N.M., Kumar S.S., Ashraf P.M., Xavier K.A.M., Prathish K.P., Ajay S.V., Edwin L., Turner A. (2023). Abandonment of fibreglass reinforced plastic fishing boats in Kerala, India, and chemical emissions arising from their burning. Environ. Monit. Assess..

[2] Rizzello G., Alayon J.P.G., Valigi E., Amadei F., Gasbarri F. Adoption of Recycled Fiberglass Distribution Network Components. Background, Pilot Projects and Future Developments. Proceedings of the IET Conference Proceedings.

[3] Yoon K., DiBenedetto A., Huang S. (1997). Recycling of unsaturated polyester resin using propylene glycol. Polymer.

[4] Kennerley J., Kelly R., Fenwick N., Pickering S., Rudd C. (1998). The characterization and reuse of glass fibres recycled from scrap composites by the action of fluidized bed process. Compos. Part A.

[5] Pickering S., Kelly R., Kennerley J., Rudd C., Fenwick N. (2000). A fluidized-bed process for the recovery of glass fibres from scrap thermoset composites. Compos. Sci. Technol..

[6] Cunliffe A.M., Jones N., Williams P.T. (2003). Recycling of fibre-reinforced polymeric waste by pyrolysis: Thermo-gravimetric and bench-scale investigations. J. Anal. Appl. Pyrolysis.

[7] Kaminsky W., Predel M., Sadiki A. (2004). Feedstock recycling of polymers by pyrolysis in a fluidised bed. Polym. Degrad. Stab..

[8] Gonçalves R.M., Martinho A., Oliveira J.P. (2022). Recycling of Reinforced Glass Fibers Waste: Current Status. Materials.

[9] Guo J., Rao Q., Xu Z. (2010). Effects of particle size of fiberglass-resin powder from PCBs on the properties and volatile behavior of phenolic molding compound. J. Hazard. Mater..

[10] Sun Z., Shen Z., Ma S., Zhang X. (2013). Sound absorption application of fiberglass recycled from waste printed circuit boards. Mater. Struct..

[11] Cozzarini L., Marsich L., Ferluga A. (2023). Innovative Thermal and Acoustic Insulation Foams from Recycled Fiberglass Waste. Adv. Mater. Technol..

[12] Rahimizadeh A., Kalman J., Fayazbakhsh K., Lessard L. (2019). Recycling of fiberglass wind turbine blades into reinforced filaments for use in Additive Manufacturing. Compos. Part B Eng..

[13] Revilla-Cuesta V., Skaf M., Ortega-López V., Manso J.M. (2023). Raw-crushed wind-turbine blade:Waste characterizationand suitability for use in concrete production. Resour. Conserv. Recycl..

[14] Revilla-Cuesta V., Faleschini F., Pellegrino C., Skaf M., Ortega-López V. (2024). Water transport and porosity trends of concrete containing integral additions of raw-crushed wind-turbine blade. Dev. Built Environ..

[15] Quadrini F. Sintering of Powders from Fiberglass Recycling. Proceedings of the ASME 2014 International Manufacturing Science and Engineering Conference.

[16] Quadrini F., Bellisario D., Santo L. (2016). Molding articles made of 100% recycled fiberglass. J. Compos. Mater..

[17] Bellisario D., Iorio L., Proietti A., Quadrini F., Santo L. (2024). Recycling of thermoset fiberglass by direct molding of ground powders. Mater. Res. Proc..

[18] Standard Test Method for Flexural Properties of Unreinforced and Reinforced Plastics and Electrical Insulating Materials by Four-Point Bending.

[19] Quadrini F., Bellisario D., Santo L., Hren I. (2013). Direct moulding of rubber granules and powders from tyre recycling. Appl. Mech. Mater..

